# OMIM.org: Online Mendelian Inheritance in Man (OMIM^®^), an online catalog of human genes and genetic disorders

**DOI:** 10.1093/nar/gku1205

**Published:** 2014-11-26

**Authors:** Joanna S. Amberger, Carol A. Bocchini, François Schiettecatte, Alan F. Scott, Ada Hamosh

**Affiliations:** 1 McKusick-Nathans Institute of Genetic Medicine, Johns Hopkins University School of Medicine, Baltimore, MD 21287, USA; 2 FS Consulting, LLC, Salem, MA 01970, USA

## Abstract

Online Mendelian Inheritance in Man, OMIM^®^, is a comprehensive, authoritative and timely research resource of curated descriptions of human genes and phenotypes and the relationships between them. The new official website for OMIM, OMIM.org (http://omim.org), was launched in January 2011. OMIM is based on the published peer-reviewed biomedical literature and is used by overlapping and diverse communities of clinicians, molecular biologists and genome scientists, as well as by students and teachers of these disciplines. Genes and phenotypes are described in separate entries and are given unique, stable six-digit identifiers (MIM numbers). OMIM entries have a structured free-text format that provides the flexibility necessary to describe the complex and nuanced relationships between genes and genetic phenotypes in an efficient manner. OMIM also has a derivative table of genes and genetic phenotypes, the Morbid Map. OMIM.org has enhanced search capabilities such as genome coordinate searching and thesaurus-enhanced search term options. Phenotypic series have been created to facilitate viewing genetic heterogeneity of phenotypes. Clinical synopsis features are enhanced with UMLS, Human Phenotype Ontology and Elements of Morphology terms and image links. All OMIM data are available for FTP download and through an API. MIMmatch is a novel outreach feature to disseminate updates and encourage collaboration.

## INTRODUCTION

Online Mendelian Inheritance in Man (OMIM), a continuation of Dr Victor A. McKusick's *Mendelian Inheritance in Man* (MIM) (1), is the primary repository of comprehensive, curated information on genes and genetic phenotypes and the relationships between them. MIM was published through 12 editions between 1966 and 1998, and OMIM has been online and searchable since 1987. Unlike databases of primary data, OMIM synthesizes and summarizes new and important information based on expert review of the biomedical literature. As a necessary outgrowth of its work, OMIM also plays a leading role in the naming and classification of genetic phenotypes. With the advent of new sequencing technologies, there is a rapid increase in the reports of presumed gene-phenotype relationships. OMIM.org was created to provide a user-friendly and easily searchable portal to a curated compilation of the literature to aid in clinical and molecular genetic research.

As of 30 October 2014, OMIM is comprised of over 22,634 entries describing 14,831 genes and 7,894 phenotypes. While OMIM content is still indexed and accessible at the National Center for Biotechnology Information (NCBI), the OMIM.org website has different indexing, greater searching capabilities, novel and user-friendly displays of gene/phenotype relationships, and topically organized links to a wide variety of external resources, which are targeted to information specifically related to data in the OMIM entry. For computational users, OMIM data are available for FTP download, and OMIM.org has a robust, state-of-the-art Application Programming Interface (API) with detailed online help documents. The API enables batch queries and computational integration of data on the fly.

## OMIM SOURCE MATERIAL

Source material for OMIM is the peer-reviewed biomedical literature. Articles are identified for inclusion into OMIM in several ways. In addition to reviewing over 45 high-impact journals, we perform targeted searches of PubMed, Current Contents and other publisher-based full-text resources. OMIM staff review genetics-related news feeds and identify articles in the process of curating information into OMIM. Our users also bring many articles to our attention. Priority is given to papers that provide significant insight into the gene-phenotype relationship, expand our understanding of human biology, or contribute to the complete clinical characterization of a disorder, disease etiology and pathogenesis. To facilitate access to additional articles on a topic that are not in OMIM, a ‘reference plus’ icon appears after each paragraph. Clicking on this icon will bring up other articles in PubMed with content similar to the references in the OMIM entry paragraph.

## OMIM STRUCTURE

**Figure 1.**
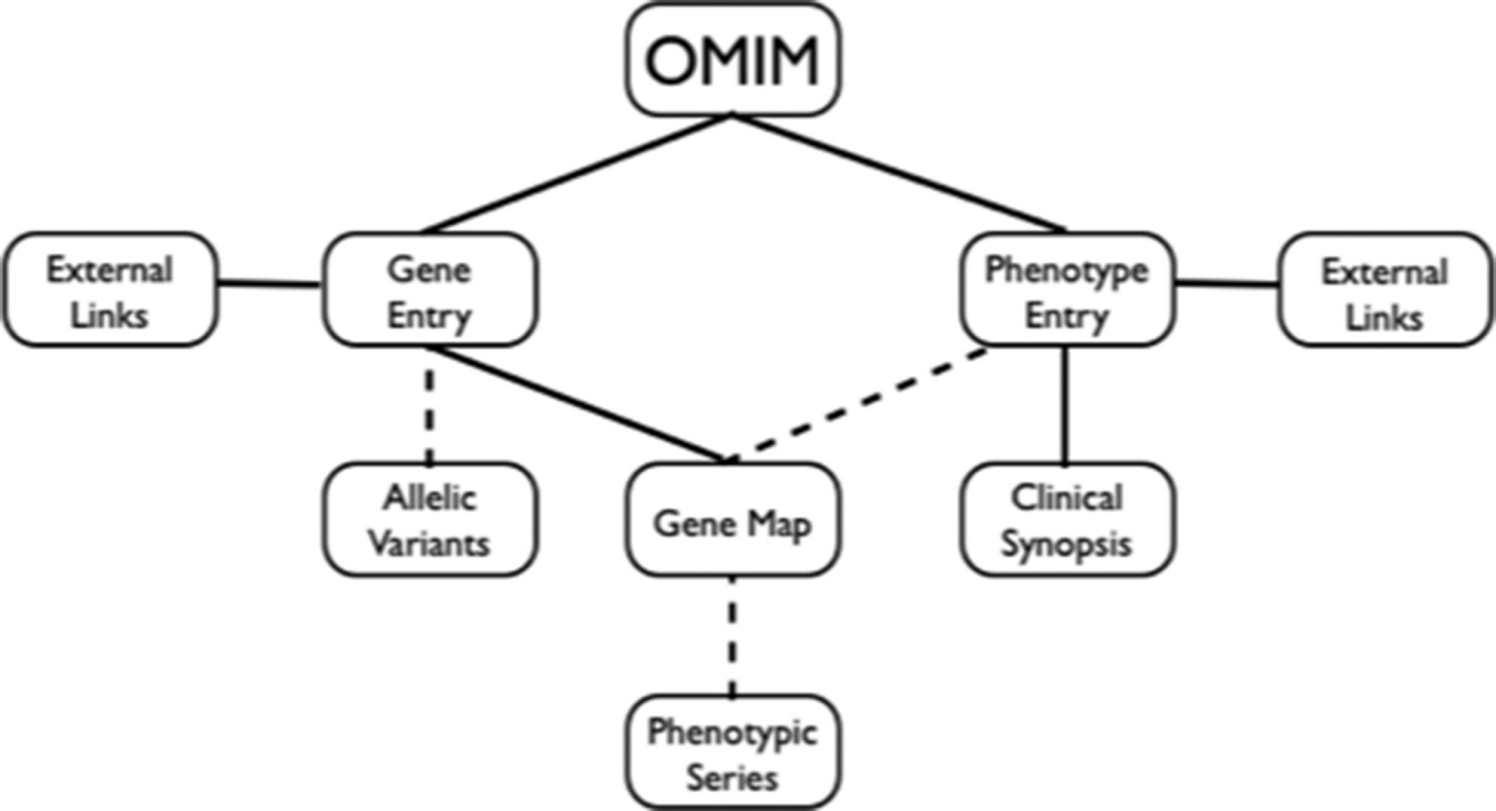
Diagram of OMIM content. Dashed lines indicate that not all genes have allelic variants; not all phenotypes are mapped; and mapped phenotypes are not necessarily part of a Phenotypic Series.

The overall structure of OMIM is shown in Figure 1. Descriptions of genes are separate from those of phenotypes because distinct mutations in one gene may cause different phenotypes. Variants in a gene reside in the gene entry. Clinical synopses, brief structured clinical descriptions, are linked to the phenotype entries. When several phenotype entries overlap significantly in their clinical manifestations, they may be curated into a Phenotypic Series. The creation of Phenotypic Series is a matter of clinical judgment, not computed similarity. OMIM's gene map is a tabular database that brings together genes and phenotypes when evidence merits and facilitates the creation of Phenotypic Series. As an adjunct to native OMIM data, OMIM gene and phenotype entries have copious external links to relevant information in other curated databases.

Both gene and phenotype entries are structured with fixed headings. At OMIM.org these headings are indexed for field-delimited searching (Table 1). (A complete list of fields and guidance on field-delimited searching is given in the online ‘Help’ link at the top of every OMIM.org page). Entries can also have subheadings; some of the subheadings are standard and appear across many MIM entries (e.g., ‘Associations Pending Confirmation’), and others are specific to an entry (e.g. ‘SNRPN Upstream Reading Frame (SNURF)’ in entry 182279). Both types of entries also have a Table of Contents (TOC) on the right side of the entry. The TOC shows the headings available in the entry. A user can scroll through the entry text or use the TOC as a quick way to access specific entry sections. The External Links for each entry are at the bottom of the TOC. These links are organized under topical headings (Table 2). The TOC, the External Links and the OMIM search query box are available at all times while scrolling through an OMIM entry.

**Table 1.** Commonly used OMIM entry headings and external reference IDs and corresponding field search term

OMIM entry heading or external reference ID	OMIM search field name
NUMBER and prefix	number or prefix
TITLE	title or ti_preferred
<Alternative title(s); symbol(s)>	ti_alternative
<Included title(s): symbol(s)>	ti_included
TEXT	Tx
DESCRIPTION	tx_description
NOMENCLATURE	tx_nomenclature
CLINICAL FEATURES	tx_clinical_features
BIOCHEMICAL FEATURES	tx_biochemical_features
INHERITANCE	tx_inheritance
CYTOGENETICS	tx_cytogenetics
MAPPING	tx_mapping
PATHOGENESIS	tx_pathogenesis
POPULATION GENETICS	tx_population_genetics
ANIMAL MODEL	tx_animal_model
CLONING and EXPRESSION	tx_cloning
GENE STRUCTURE	tx_gene_structure
GENE FUNCTION	tx_gene_function
MOLECULAR GENETICS	tx_molecular_genetics
GENOTYPE/PHENOTYPE CORRELATIONS	tx_genotype_phenotype_correlations
EVOLUTION	tx_evolution
ALLELIC VARIANTS	Av
<Allelic variant number>	Av_number
<Allelic variant title>	Av_name
<Allelic variant included title>	Av_alternative_names
<Allelic variant mutation>	Av_mutations
REFERENCES	Ref or ref_author or ref_pubmed_id
CONTRIBUTORS	contributors and creator
EDIT HISTORY	date_updated and date_created
	
Reference DNA sequence ID	ncbi_reference_sequence
Swiss Prot ID	swiss_prot_id
Ensembl transcript ID	ensembl_id
Mouse Genome Informatics gene ID	mgi_id

Note: For example, to search for muscular dystrophy in OMIM entry titles, enter the following within the search box: title: ‘muscular dystrophy’. The clinical synopses also support field-restricted searching. A complete list of the OMIM search fields is available from the online search help section 1.5.

**Table 2.** OMIM external links

**Genome**	**Clinical**	**Variation**	**Animal models**
Ensembl	ClinicalTrials.gov	ClinVar	FlyBase
MITOMAP	DECIPHER	Genetics association DB	HomoloGene at NCBI
NCBI map viewer	EuroGentest	GWAS central	MGI mouse phenotype
UCSC	GARD	HGMD	KOMP
**DNA**	Gene tests	HGVS	IKMC
Ensembl	Gene reviews	Locus-specific DBs	MGI mouse gene
NCBI RefSeq	Genetic alliance	LOVD	Wormbase gene
UCSC	GTR	Insight	ZFin
**Protein**	Newborn screening	NHLBI EVS	OMIA
HPRD	NextGxDx	1000 Genomes	**Cell Lines**
UniProt	OrphaNet		Coriell
**Gene Info**	POSSUM		**Pathways**
BioGPS			KEGG
Ensembl			Reactome
GeneCards			
Gene Ontology			
HGNC			
KEGG			
NCBI gene			
PharmGKB			
UCSC			

Note: Links to external resources take users to the topic-specific information and are grouped into categories (bold headings) that appear in an OMIM entry's Table of Contents. Links will not appear if there is no relevant information in the external resource. A description of these resources is available from the link at the top of every OMIM.org page.

OMIM numbers are widely used in the biomedical literature and in many databases. Each OMIM entry has a unique six-digit number. Autosomal entry numbers start with a 1, 2 or 6 (for entries created after 15 May 1994). X-linked entries start with a 3, Y-linked entries with a 4 and mitochondrial entries with a 5 (see online FAQ 1.2). Allelic variants (AVs) are designated by the MIM number of the entry, followed by a decimal point and a unique 4-digit variant number (e.g. Hemoglobin S is designated 141900.0243).

**Figure 2.**
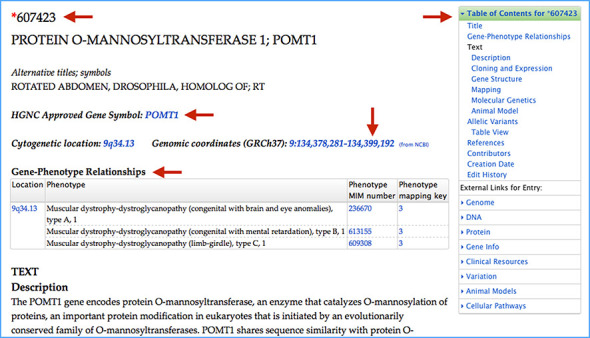
OMIM entry for protein O-mannosyltransferase 1 (607423). Arrows call attention to the following: the unique MIM number 607423 with an asterisk prefix denoting a gene. Approved gene symbol obtained directly from the Human Gene Nomenclature Committee data. Cytogenetic location and Genomic coordinates obtained from NCBI. Gene–phenotype relationships table showing allelic disorders, their MIM numbers, and the phenotype mapping key. The Table of Contents (TOC) facilitates navigation within the OMIM entry, and external resource links specific for the gene are topically organized and listed below the TOC.

Gene entries in OMIM are distinguished by an asterisk before the MIM number. A small (91) but diminishing number of entries are combined gene and phenotypes and have a plus sign before the MIM number. Gene entries describe protein-coding genes, regulatory elements, micro-RNAs, non-coding RNAs and other functional elements as they are recognized (Figure 2). Discussion in these entries may include information on gene structure, isoforms, expression, function, crystal structure, molecular genetics, imprinting, methylation and animal models. Many entries have markup language and unique text elements to tag specific information and link it to resources (e.g. MIM numbers, sequence accession numbers, dbSNP numbers (2)). If there are variants in the human gene that lead to a phenotype, the gene will have an AV section, which includes only selected variants as described below.

**Figure 3.**
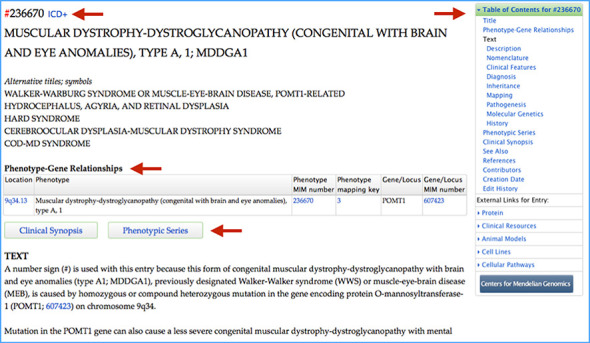
OMIM entry muscular dystrophy-dystroglycanopathy type A1. Arrows call attention to the following: The MIM number for this entry is preceded by the prefix # denoting a phenotype that has a known molecular basis. A link for the ICD+ codes is available for this entry. The phenotype–gene relationship table shows the phenotype (with MIM number) followed by the gene (with MIM number) that is mutated in the phenotype. When available, links to OMIM's clinical synopses and phenotypic series are located in the phenotype entry. The TOC facilitates navigation within the entry, and links to external resources specific for the phenotype are available below the TOC.

Phenotype entries in OMIM are set apart by the MIM number prefixes # (molecular basis known), % (mendelian, but no molecular basis yet found) or null (possibly mendelian, but no known inheritance pattern or molecular basis). Phenotypes (a set of observable characteristics of an organism) in OMIM include single gene mendelian disorders (e.g. cystic fibrosis, sickle cell disease, achondroplasia), phenotypic traits (e.g. hair and eye color, PTC tasting), susceptibility to drug reaction (e.g. malignant hyperthermia, warfarin sensitivity), altered susceptibility or reaction to infection (e.g. herpes simplex encephalitis, progression of HIV infection to AIDS), germline susceptibility to cancer (e.g. BRCA1/2 and breast/ovarian cancer) and recurrent deletion and duplication syndromes (17p11.2 deletion and duplication syndromes). A phenotype entry will include, as available, description of the essential clinical features, patient and family reports, inheritance pattern, cytogenetic and molecular genetic findings, and links to OMIM's clinical synopsis and/or phenotypic series. Phenotype entries may display ICD or SNOMED CT (3, 4) codes when available (Figure 3).

## CLINICAL SYNOPSES

The OMIM clinical synopses are tabular representations of the phenotypic features of a disorder and are organized anatomically with fixed headings and subheadings. They are created primarily for clinical geneticists for use in the clinic. The features under the headings are taken from the literature and incorporated into the synopsis using a semi-controlled vocabulary. Many features include modifiers and additional terminology specific to medical subspecialities that are helpful for delineating overlapping disorders and distinguishing characteristic features. Among genetically heterogeneous disorders, care is taken to include only those features that are present in patients with mutations in the same causative gene.

**Figure 4.**
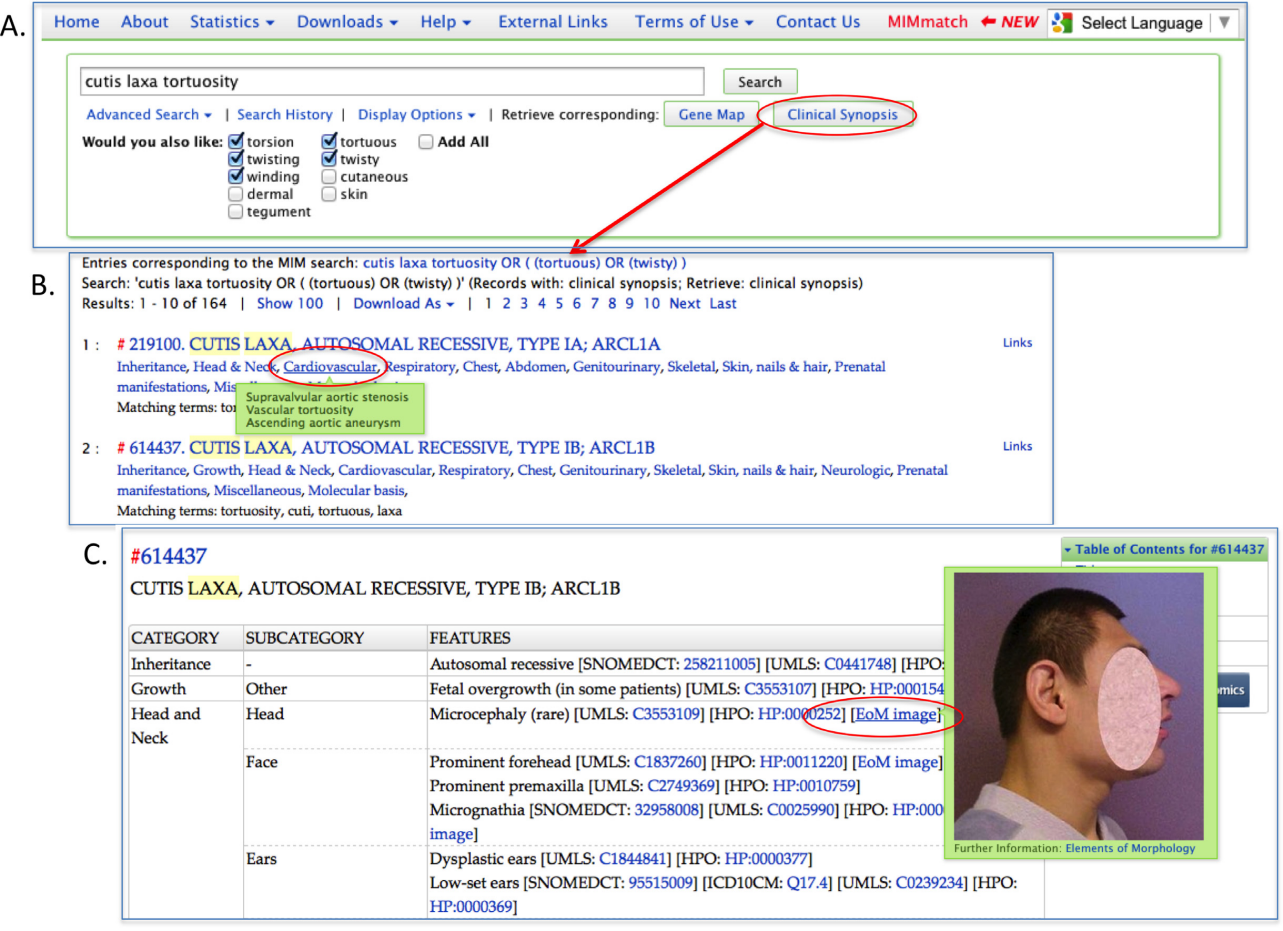
(**A**) OMIM search navigation box showing optional thesaurus terms for search terms tortuosity and cutis. Access to the clinical synopsis quick view for these entries is available from the Retrieve Corresponding Clinical Synopsis button. (**B**) Clinical synopsis quick view showing anatomical categories and mouse-over function to reveal detailed features. (**C**) Clinical synopsis of OMIM entry 614437 showing the optional display of clinical identifiers to the UMLS, SNOMED CT, HPO and EoM. A mouse-over of the EoM image link shows the image of the phenotypic feature (in this case, microcephaly). Users can search these identifiers through the OMIM.org website or through the OMIM.org API.

In OMIM.org, OMIM's clinical synopses have a new look and new search options. In addition to searching on the text of the synopses, a user can search for synopses that have features in any of the major anatomical headings. To help users compare features across multiple entries, OMIM.org has a clinical synopsis quick view (Figure 4). This optional view is easily accessible by selecting the ‘Retrieve corresponding: Clinical Synopsis’ button available after any search of OMIM. To assist in the computational mapping of clinical features across platforms and programs, clinical features in OMIM.org are mapped to unique identifiers in the Unified Medical Language System (UMLS) (5), Human Phenotype Ontology (HPO) (6), SNOMED CT (3, 4) and the Elements of Morphology (EoM) (7). OMIM and SNOMED CT have been UMLS source vocabularies for decades and as such are mapped into the UMLS by the National Library of Medicine biannually. Mapping of OMIM clinical synopsis terms to HPO and EoM is both a computational and manual curation process. These mappings are now viewable from a toggle button in the clinical synopsis viewer and are also available through the OMIM API. Images from the Elements of Morphology are viewable from a mouse-over next to many features in OMIM's clinical synopses (Figure 4).

## THE MORBID AND SYNOPSIS MAPS

OMIM's gene map is a distillation and tabular representation of the information in OMIM with a focus on the ‘morbid anatomy of the human genome’ and is organized by gene or locus and chromosomal location. The tabular layout clearly displays the relationships between gene and phenotype and highlights the placement of human disease on the genome. Every phenotype on the map is assigned a phenotype mapping key numbered 1–4: (1) the disorder was positioned by mapping of the wild-type gene; (2) the disorder itself was mapped; (3) the molecular basis of the disorder is known; or (4) the disorder is a chromosome deletion or duplication syndrome. These mapping keys are explained in the FAQ as well as from a mouse-over of the number.

**Figure 5.**
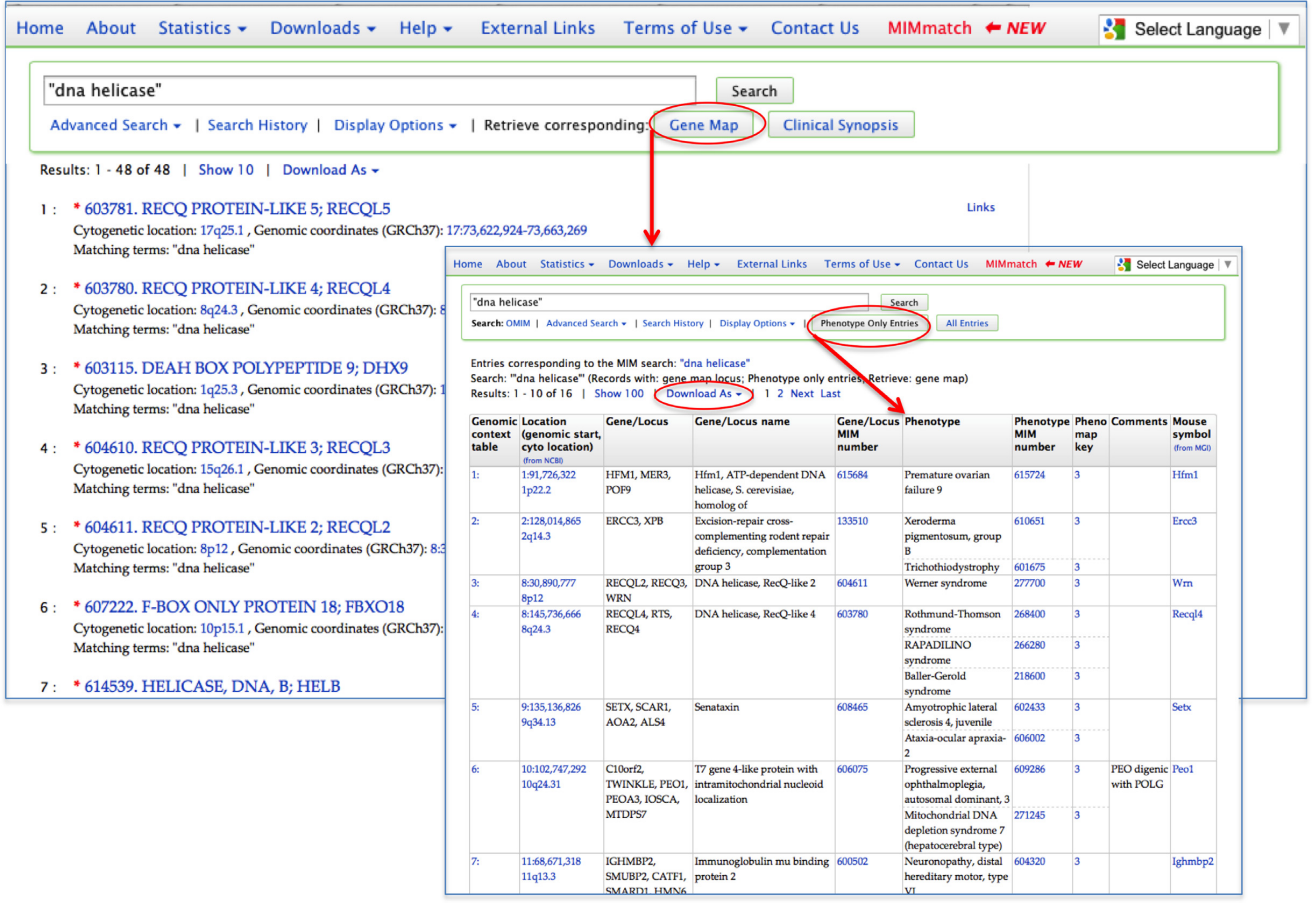
OMIM gene map. OMIM.org provides a unique way to access gene map information from an OMIM retrieval set from the Retrieve Corresponding Gene Map button. From within the gene map retrieval set, a user can view only those entries in the map that have phenotypes by selecting the Phenotype Only Entries. A retrieval set can be downloaded as an Excel or tab-delimited file by selecting the Download As option.

OMIM.org now facilitates accessing gene map data that correlates to a search of OMIM. For example, a search of OMIM for ‘dna helicase’ (with quotes) retrieves 48 entries in OMIM. To view the genes and loci associated with this retrieval set, select ‘Retrieve corresponding: Gene Map’ from the OMIM search and navigation box. To see the phenotypes associated with this Gene Map retrieval set, select ‘Phenotype Only Entries’ to reveal the resulting 16 genes with 33 phenotypes among them (Figure 5).

To facilitate finding phenotypes that are located within a genomic coordinate range, OMIM.org provides a unique search tool to query the Morbid Map by genomic coordinate (currently build GRCh37). This is available from the Advanced Search-Gene Map page link located below the main OMIM search box. A search of OMIM's gene map using the genomic coordinates chr2:164,744,057–166,737,379 will retrieve the 16 genes and loci that fall within or overlap the coordinates in the query. To see just the entries with phenotypes, select the ‘Phenotype Only Entries’ button, which shows that there are 12 genes with 14 phenotypes located in the region. Genomic coordinate searching is explained on the Gene Map Advanced Search page.

## REPRESENTATION OF GENE–PHENOTYPE RELATIONSHIPS

**Figure 6.**
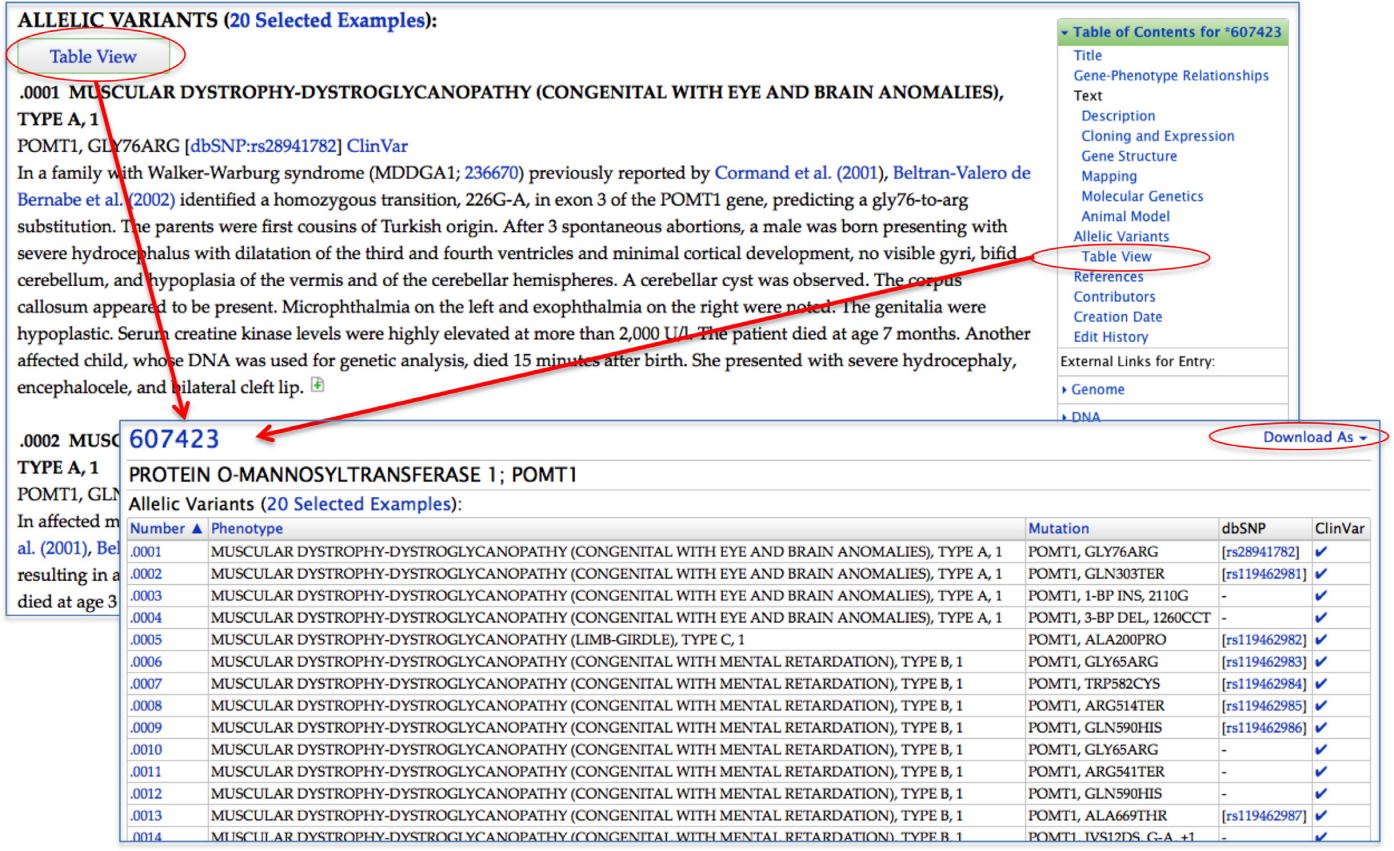
Allelic variants. Allelic variants are included in gene entries. The full view of the variant includes mutation details, clinical features of patients with the mutation. The Table View of the variants can be accessed from a link below the Allelic Variants heading or from the TOC. The Table View information can be downloaded as an Excel or tab-delimited file.

AVs are at the core of gene-phenotype relationships in OMIM. Currently, there are over 22,000 AVs in OMIM. For most genes, only selected mutations are included. Criteria considered for inclusion are the first mutation to be discovered, high population frequency, distinctive phenotype, historic significance, unusual mechanism of mutation, unusual pathogenetic mechanism or distinctive inheritance (e.g. dominant with some mutations and recessive with other mutations in the same gene). Most of the AVs represent disease-causing mutations, but a few polymorphisms are included, many of which show a positive correlation with particular common disorders. Each AV has an OMIM variant number, title, mutation and a text section that provides a complete description of the mutation with supporting data and pertinent information about the disorder and affected individuals as provided in the cited article. A Table View option of the AVs provides a quick view of this information without the associated text. Variant titles and mutation fields are indexed for searching, and, when available, links to dbSNP (2) and ClinVar (8) are provided (Figure 6).

The variants included in OMIM have been ascertained by a variety of technologies and the certainty of the reported gene-phenotype correlation is variable. Given the rapid identification of variants provided by next-generation sequencing, as of January 2013, OMIM applies the following criteria for establishing a gene-phenotype relationship: (i) the existence of multiple, unrelated individuals with pathogenic variants in the same gene; (ii) the variants segregate with the phenotype in multiplex families; and/or (iii) the variants occur *de novo* in a statistically significant number of individuals. A qualified gene-phenotype relationship is established based on the following: (i) only 1 multiplex family is reported to have variants in a single gene and the variants segregate with the phenotype in the family and (ii) there is supportive functional data such as a comparable phenotype in a model organism, *in vitro* or *in vivo* enzyme or gene activity experiments, mutation location in a conserved region or functional pathway support. Recognizing the rarity of some phenotypes, a gene-phenotype relationship may be established on the basis of a single patient if there is robust supporting phenotype and functional data. When a gene-phenotype relationship is established based on a single family or patient, the variant title is followed by ‘1 family’ or ‘1 patient.’ When AVs are thus labeled, the phenotype in the Morbid Map is preceded by a ‘?’ with appropriate notation in the comments field. These qualifiers are removed when additional supporting information is added to the entry. If, after extensive consideration of a paper reporting a novel gene-phenotype relationship, a variant does not meet these criteria, the variant may be included in the gene entry with a title of ‘variant of unknown significance.’ Occasionally, new published research on a variant may call into question that variant's relationship with a disorder. For these cases, OMIM ‘reclassifies’ the variant and places an explanation for the reclassification at the top of the variant. Examples of these variants may be found by searching OMIM.org for ‘av_name:family’ or ‘av_name:patient’ or ‘av_name:significance’ or ‘av_name:reclassified.’ This policy is being applied retrospectively to existing variants in OMIM as part of ongoing curation efforts.

To provide a straightforward view of the correlation between gene and phenotype, we have placed gene–phenotype (Figure 2) and phenotype–gene (Figure 3) relationship tables near the top of the gene and phenotype MIM entries, respectively. This allows quick ascertainment of whether a gene is known to be involved in disease and the degree of phenotypic diversity at that gene.

## OMIM PHENOTYPIC SERIES

**Figure 7.**
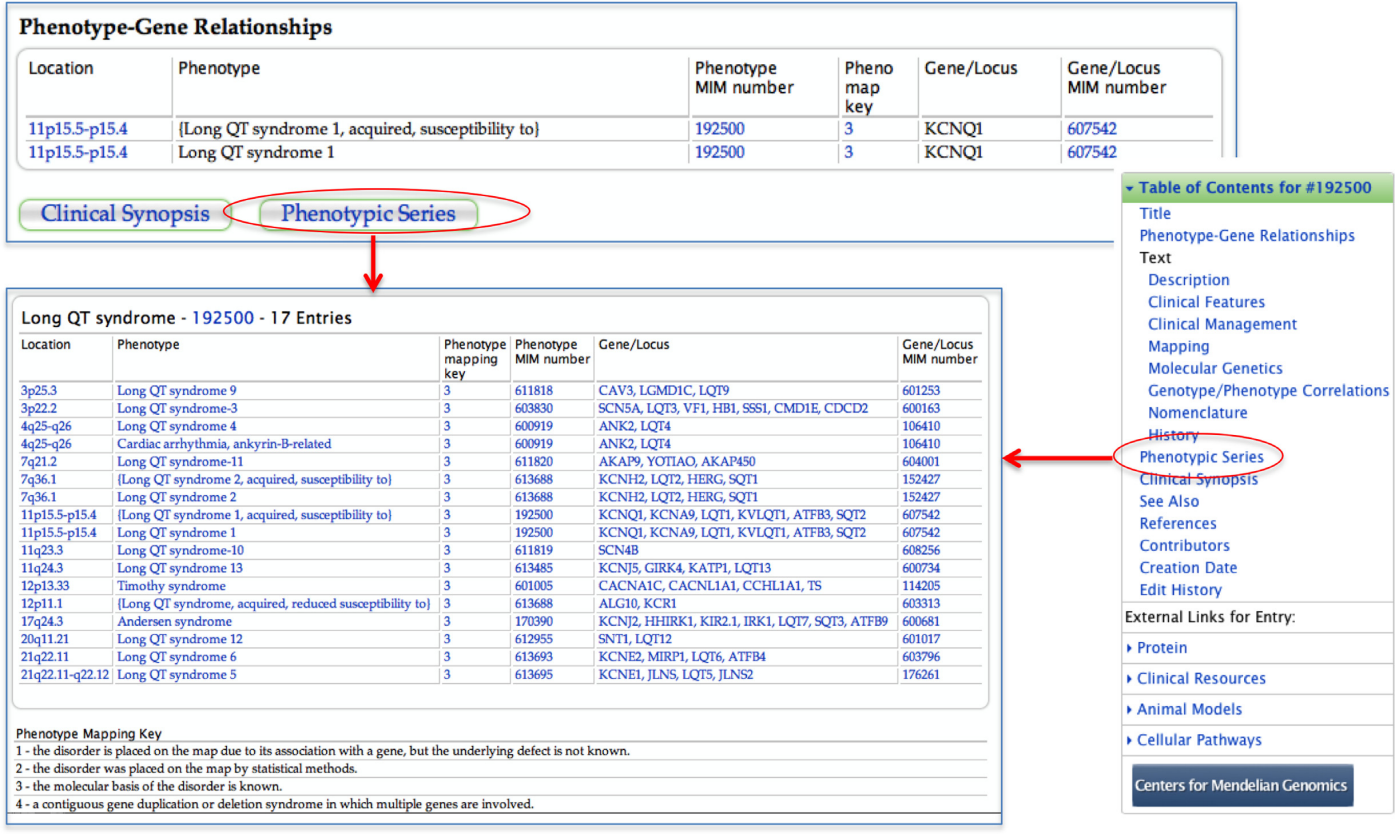
OMIM Phenotype Series can be accessed from within phenotype entries from a link below the phenotype–gene relationship table or from the TOC. A list of disorders with a Phenotypic Series is available from the FAQ or through MIMmatch.

A Phenotypic Series is a tabular view of genetic heterogeneity of identical or similar phenotypes across the genome. The series are generally created when different mapping or a new causative gene is identified for what is considered to be a well-defined mendelian phenotype (9). The series are created from a nosology perspective and may therefore change over time as our knowledge of a disorder or class of disorders grows. A phenotype may be included in more than one Phenotypic Series (e.g. entry 613157, which is an autosomal recessive limb-girdle muscular dystrophy and a type of muscular dystrophy-dystroglycanopathy). If a phenotype is included in a series, a link to the series is in the TOC of the phenotype as well as below the phenotype–gene relationship table (Figure 7). To date, OMIM has 315 different phenotypic series comprised of 2528 phenotypes. A list of the phenotypes that are in series is available from within the FAQ information from the link at the top of every OMIM.org page.

## SEARCH FUNCTIONS AND OUTPUT

Apache Lucene Solr™, an open-sourced enterprise search platform, is used as the full-text search engine to handle all searches on OMIM.org. Searches are augmented with spellcheck, autofill, proximity searching and thesaurus matching. Rich vocabulary search support files have been added that include nonstandard plurals (fibula, fibulae), British to American English mappings (e.g. oesophagus, esophagus), synonyms (patella and kneecap) and noun-adverb support (atrial, atrium). A user-directed thesaurus provides the ability to include similar terms in a search at a user's discretion. For example, the search term ‘dwarf’ will offer the additional terms ‘growth retardation,’ ‘hyposomia’ and ‘short stature.’ The retrieval list notes which search terms matched in each entry, and the search terms are highlighted within the entry. Double-clicking on a word in OMIM.org will pop up a ‘Define’ link that will take the user to a collection of definitions of the word at medical-dictionary.thefreedictionary.com. Using double quotes around search terms provides proximity searching and extends the precision of retrievals beyond simple Boolean matches. For example, to retrieve entries that are relevant to cytokine receptors, a user can search on ‘cytokine receptor’ (using double quotes) and retrieve 127 OMIM entries rather than searching on cytokine AND receptor, which would retrieve 790 entries.

An Advanced OMIM search page provides quick ways to search for entry number prefixes or to restrict searching within entries, e.g. in AVs. A Search History page provides an easy way for a user to keep track of previous searches or to combine searches. Finally, OMIM.org now features Excel and tab-delimited output options for search retrieval sets from OMIM and the gene map.

## OMIM FTP SERVICE

OMIM provides several files for FTP download. Registration for FTP access is made from the ‘Downloads’ link at the top of every OMIM.org web page. An email acknowledging the registration is sent with instructions and tips on using the FTP service. The registration process also provides us with necessary information to contact users for recommendations for improvements to the FTP service. The files currently provided include:

*mim2gene.txt*—a field delimited table of OMIM numbers matched to NCBI gene IDs. This table can be used to map OMIM entries to many other resources that use NCBI Gene IDs or NCBI data that may be mapped to NCBI Gene IDs.
*omim.txt.Z*—a compressed file of all native OMIM data. Data from other resources that is revealed on the website is available through the API.
*genemap, genemap2.txt, genemap.key*—a field delimited file of OMIM's synopsis gene map and a key to the fields in the map. The genemap version of the map is arranged by chromosome, 1pter through Yqter and each record has the gene or locus name and MIM number and, if available, any disorders associated with the gene along with the phenotype mapping key for the disorder. Allelic disorders appear in one field separated by a semicolon.
*morbidmap*—a field-delimited file that is a subset of records from the genemap, parsed and sorted alphabetically by disorder.

## OMIM API SERVICE

**Figure 8.**
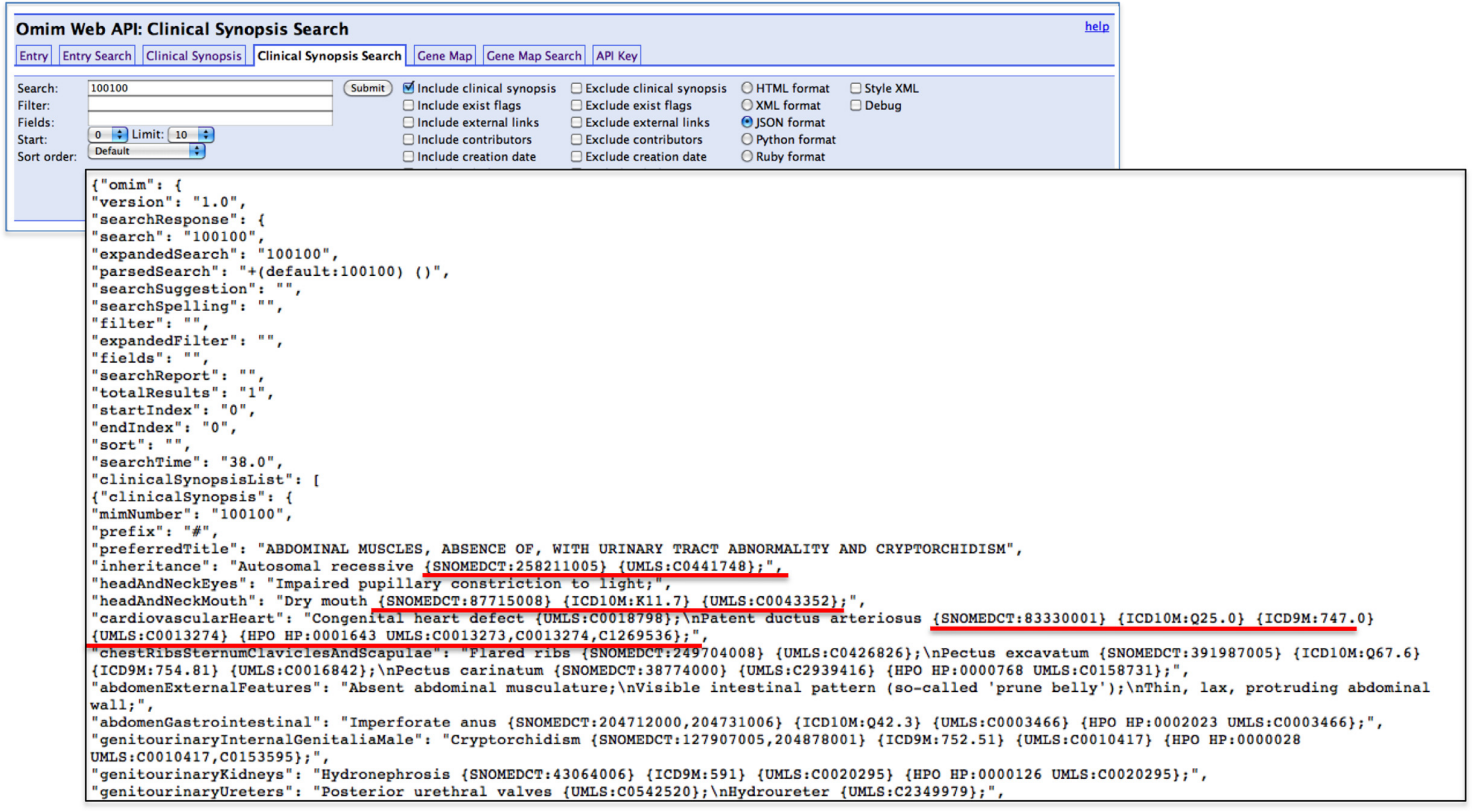
Example of API output of an OMIM clinical synopsis. For this example, the output format is JSON and examples of the unique identifiers for UMLS, HPO identifiers are underlined. The API enables batch queries of all OMIM data and allows computational integration of data on the fly.

The OMIM API is a web-based REST API that responds to requests made over HTTP. Registration for the API is available from the Downloads link at the top of every OMIM.org page. An email is sent to users with their API key and instructions. The ‘Help’ link at the top of every OMIM.org page provides access to a concise and instructive API-specific guide. Because the OMIM website is built on the API, most of the functionality that is available on the website is also available in the API. The API accepts a wide variety of requests, including searching for entries and fetching individual entries or lists of entries. Results can be fetched in batches. Users can control which entry text sections they want to download, such as only references, only AVs, only text subsections (e.g. titles, descriptions), etc. The gene map can be searched using text, location, genomic coordinates, or chromosome. The API also offers a very simple web browser-based user interface in which users can enter a variety of parameters and see the results in whatever format they choose (Figure 8). This is an invaluable tool to test requests and to see how the response is formatted. The API output is available in a variety of formats including XML, JSON, JSONP, Python and Ruby.

## OMIM UPDATES AND STATISTICS

Following the progress in OMIM of heritable disorders or particular genetic topics of interest is easier than ever. From the Statistics link at the top of every OMIM page, a user can view the progress in growth of MIM entries (OMIM Entry statistics), the progress of disease gene discovery (OMIM Gene Map Statistics) or the actual daily updates to OMIM (Update List). The OMIM Update List shows separately the MIM numbers and titles of all new entries, updated entries, new clinical synopses, and updated clinical synopses by day. These data are then organized by month and year. Users can subscribe to an RSS feed from this page. Within a MIM entry, all new or updated information is marked to the left of the text by green side bars. These bars are retained for three months.

## MIMmatch and COMMUNITY ENGAGEMENT

A new OMIM service, MIMmatch, was instituted in late 2013 that allows users to (i) designate entries they wish to follow and to receive email alerts when the entries are updated; (ii) find other researchers who may share their interest in certain entries; (iii) receive a daily update on any new gene-phenotype relationships established in OMIM; and/or (iv) follow updates to phenotypic series. There are currently over 600 registered MIMmatch users. The names of MIMmatch users will not be shared with any third party, and no more than one e-mail notification per day is sent. Users need to register with a valid e-mail address and confirm the registration.

OMIM engages with the clinical and molecular genetics community through online help and tutorials, user surveys, focus groups and lectures. The Contact Us link at the top of every OMIM.org page is the primary way for users to submit comments, questions, corrections or suggestions. Users primarily suggest new references to add to OMIM but also request technical and search help. OMIM staff worked with OpenHelix™ to create an online OMIM tutorial with dowloadable exercises, slides, and handouts. Access to this is available from OMIM's home and FAQ pages. Recently, a recorded overview of OMIM given as a lecture for the NCI Center for Biomedical Informatics and Information Technology Speaker Series was made available (https://www.youtube.com/watch?v=s12ZQSbkTO8).

## USE OF OMIM DATA IN OTHER RESOURCES

In addition to the long-standing use of OMIM terminology as source data within the UMLS, OMIM data are now used as source material for many other databases. For example, OMIM clinical synopses formed the foundation for the HPO terminology, which continues to incorporate new information from OMIM. OMIM gene-phenotype relationships are integrated in many genome interpretation tools, including Phevor (10), Exome Walker (11) and PhenoDB (12). OMIM AVs are incorporated into ClinVar. OMIM data are the basis for the human-mouse disease connection resource at the Mouse Genome Informatics resource. OMIM data have also been used to support a single-nucleotide polymorphism (SNP) array evaluation tool that facilitates identifying potential recessive disease genes in regions of homozygosity in children of consanguineous unions (13). We welcome collaborative efforts in the use of OMIM in research.

OMIM has been an essential resource for clinicians and researchers in molecular biology, genetics and genomics for nearly 50 years. Through its careful selection, review and curation of the scientific literature, it maintains a current, authoritative source of information on the evolving knowledge of the relationship between genes and disease. Its free-text, structured format provides the flexibility necessary to explain the nuances of these relationships as well as to describe newly identified biological and pathological processes underlying them. As genomics becomes more integral to all fields of medicine, the unparalleled breadth and richness of description of human phenotypes and genes in OMIM will provide expert and timely support to clinicians and researchers in diverse scientific fields.
